# Continuous Flow Photochemistry for the Preparation of Bioactive Molecules

**DOI:** 10.3390/molecules25020356

**Published:** 2020-01-15

**Authors:** Mara Di Filippo, Cormac Bracken, Marcus Baumann

**Affiliations:** School of Chemistry, University College Dublin, Science Centre South, Belfield, Dublin 4, Ireland; mara.difilippo@ucdconnect.ie (M.D.F.); cormac.bracken@ucdconnect.ie (C.B.)

**Keywords:** continuous flow chemistry, photochemistry, photocatalysis, bioactive molecules, medicinal chemistry, process development, enabling technologies

## Abstract

The last decade has witnessed a remarkable development towards improved and new photochemical transformations in response to greener and more sustainable chemical synthesis needs. Additionally, the availability of modern continuous flow reactors has enabled widespread applications in view of more streamlined and custom designed flow processes. In this focused review article, we wish to evaluate the standing of the field of continuous flow photochemistry with a specific emphasis on the generation of bioactive entities, including natural products, drugs and their precursors. To this end we highlight key developments in this field that have contributed to the progress achieved to date. Dedicated sections present the variety of suitable reactor designs and set-ups available; a short discussion on the relevance of greener and more sustainable approaches; and selected key applications in the area of bioactive structures. A final section outlines remaining challenges and areas that will benefit from further developments in this fast-moving area. It is hoped that this report provides a valuable update on this important field of synthetic chemistry which may fuel developments in the future.

## 1. Introduction

The last decade has witnessed a much-welcomed renaissance and subsequent exploitation of photochemical transformations within the chemistry community. This renewed interest in utilizing light to bring about chemical reactions is largely fueled by a desire to realize more sustainable approaches for target molecule synthesis, along with significant advances in the field of photoredox catalysis where both transition metals and organic dyes have been exploited. The availability of suitable light sources, ranging from light-emitting diodes (LED) to continuous fluorescent lamps (CFL) and powerful UV lamps (e.g., medium-pressure mercury lamp), furthermore, has driven this development. Consequently, a plethora of known and newly developed photoreactions has been reported in the literature, rendering facile access to diverse chemical entities through selective transformations that oftentimes offer orthogonal approaches to the synthetic chemist [1,2,3,4,5,6].

In addition, continuous flow technology [7,8,9,10,11,12,13] has had a major impact on popularizing photochemical reactions, as it not only provides the tools to effectively perform photochemical reactions, but moreover helps overcoming limitations that are inherent to photochemistry. As such, the short path lengths provided by narrow, light-permeable tubing ensure that light can easily penetrate the substrate solution, thereby mitigating limitations commonly arising from the Beer–Lambert law. Furthermore, by placing the light source near the reactor coil or microchip of the flow set-up, very efficient and uniform radiation of the substrate can be achieved. Oftentimes low-energy, tunable light sources are favored in combination with suitable cooling mechanisms to effectively control both temperature and energy input. Finally, continuous flow processing allows for simple reaction scale-up of the resulting photoreaction without alteration of reaction parameters and the short residence times within the photoreactor reduce the likelihood of decomposition of substrate or product due to over-radiation. 

As a consequence of the successful union of flow reactor technology with modern photochemical applications, a growing body of literature has emerged, including topical reviews by leading experts in the field [14,15,16,17]. As the field of continuous flow photosynthesis is moving forward at fast pace, we wish to provide a focused review on its impact on generating bioactive molecules [18,19,20]. This appears both timely and paramount, as the last ten years have provided the foundations to now apply continuous photochemistry to the syntheses of various biologically active entities—drugs, natural products and their precursors. In addition, we wished to evaluate whether there had been significant uptake of this technology by chemists outside traditional academic laboratories and whether flow photochemistry had advanced to be a viable option to generating bioactive molecules in industrial settings. To accomplish this, we first review the design of various reactor types and outline innovative aspects that aid in overcoming traditional bottlenecks. The second section investigates progress on continuous photochemical approaches targeting greener and more sustainable synthesis means. That is then followed by a dedicated section reviewing recent applications of photochemical transformations to enable the formation of bioactive species, before an assessment of the feasibility of readily integrating photochemical transformations into advanced multistep sequences in flow mode. 

## 2. Reactor Design and Technology

The diminishing penetration depth of light into solutions of substrates is one of the major challenges that has prevented effective exploitation of photochemistry in research settings. This phenomenon, described by the Beer–Lambert law, means that photochemical batch processes that typically suffer from poor mass transfer cannot be scaled effectively. Consequently, most batch photo-reactors (e.g., Rayonet reactor) use a set of lamps closely arranged around the reaction vessel to maximize irradiation. Alternatively, a single light source may be placed at the center of a double jacketed reaction vessel to irradiate the solution in an inverse arrangement. In both cases, appropriate cooling mechanisms are vital to ensure effective dissipation of the heat generated by the lamps.

The superior performance of continuous flow reactors over batch systems arises from miniaturization of mixing and reactor elements that results in excellent mass and heat transfer. Therefore, chemists and chemical engineers have taken inspiration from flow reactor designs to address the aforementioned challenges regarding photochemical reactors. Consequently, several diverse and effective flow reactor designs have been reported over the last decade and applied to different photochemical transformations [21].

**Figure 1:**
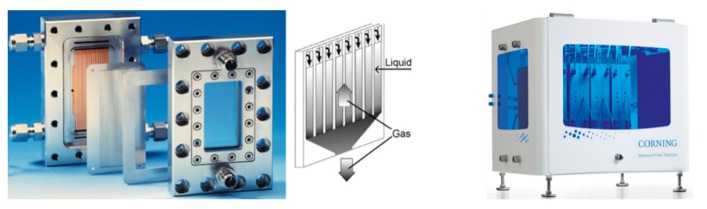
Images of a falling film reactor plate (reproduced with permission from [25]) and the Corning G3 photoreactor [26].

An example by Oelgemöller and coworkers [22] demonstrates a falling film reactor set-up to bring about the effective photooxygenation of α-terpinene in the presence of Rose Bengal to generate ascaridole (Figure 1). More recently, the Corning reactor system that is based on irradiated reactor plates has gained popularity as it allows one to effectively perform continuous photochemical reactions at various scales. In this system, panels of LEDs are arranged next to mesofluidic reactor plates, allowing for uniform radiation of the substrate solution. Published examples include the photooxidation of methionine, α-terpinene or citronellol in the presence of organic dyes (e.g., Rose Bengal, methylene blue or tetrakis(4-carboxyphenyl)porphyrin) [23,24].

**Figure 2:**
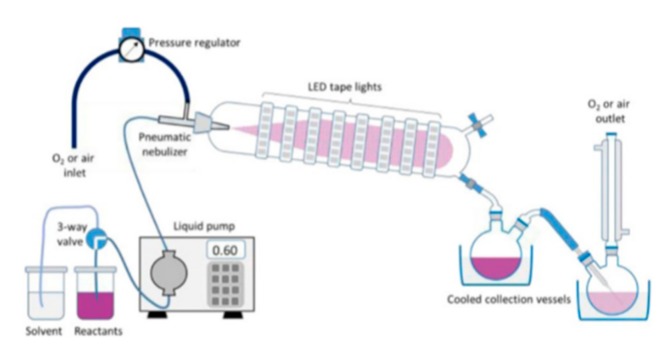
Image of the nebulizer-based photo-reactor (reproduced with permission from [27]).

Another approach to realize such photooxygenation reactions in a continuous manner was reported by Pergantis and Vassilikogiannakis, who developed a nebulizer-based photo-reactor [27]. Therein, an aerosol is generated and sprayed into an irradiated reaction chamber by mixing a stream of air or oxygen with the substrate solution. Low-energy LED strips are mounted around this chamber providing uniform radiation of the aerosol, which upon condensation is collected at the outlet of this set-up (Figure 2).

**Figure 3:**
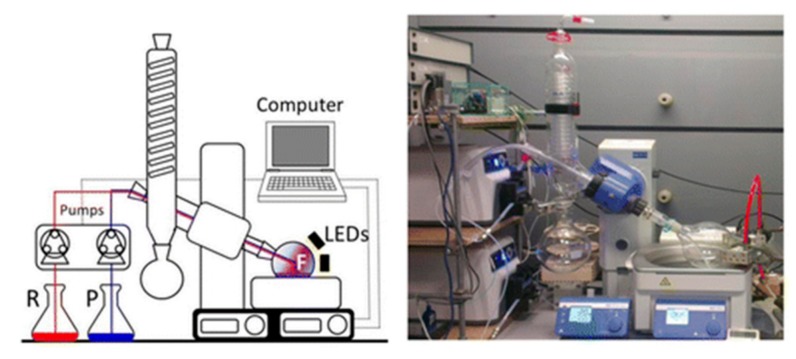
Image of the photochemical reactor set up (reproduced with permission from [28]).

Related applications that also exploit thin films were recently reported by Poliakoff and George who used a modified rotary evaporator set up (Figure 3). In this case substrate solutions are delivered into the rotating flask via the side port of the set-up. LEDs or UV–Vis lamps effectively irradiate the film generated rendering a system that can be run either continuously or semi-continuously based on low budget components that are readily available in research laboratories [28].

**Figure 4:**
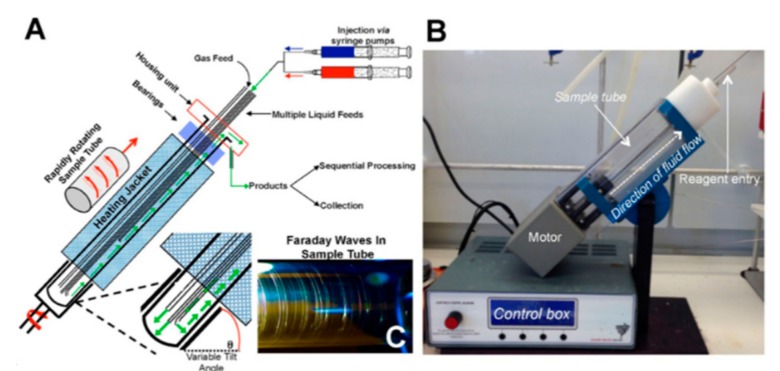
Representations of the vortex fluidic device. (**A**) A schematic of the vortex fluidic device; (**B**) A photograph of the vortex fluidic device; (reproduced with permission from [30]).

Raston and Stubbs recently reported on a vortex fluidic device that comprises a temperature-controlled reactor zone into which the substrate solution can be delivered continuously (Figure 4). The resulting thin film again provides a high surface-to-volume ratio, and thus enables effective and uniform irradiation. This was effectively demonstrated for the generation of new C–C bonds via photo-redox transformations [29].

**Figure 5:**
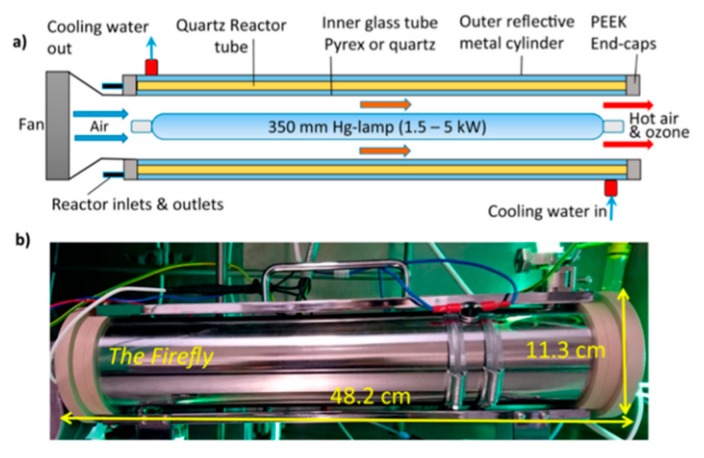
Representations of the firefly photoreactor. (**a**) Diagram of reactor; (**b**) Firefly reactor in operation; (reproduced with permission from [31]) .

In order to enable large scale generation of desired photo-products, Booker-Milburn and co-workers recently reported on a new photo-flow reactor known as Firefly (Figure 5). In this set-up, several parallel tubes are interconnected and arranged around a powerful light source (e.g., 400 W Hg-lamp). Equipped with an effective cooling mechanism, this reactor provides an internal volume of 120 mL and can deliver multigram quantities of photo-adducts per minute, which can be scaled to kilogram quantities per day [31].

**Figure 6:**
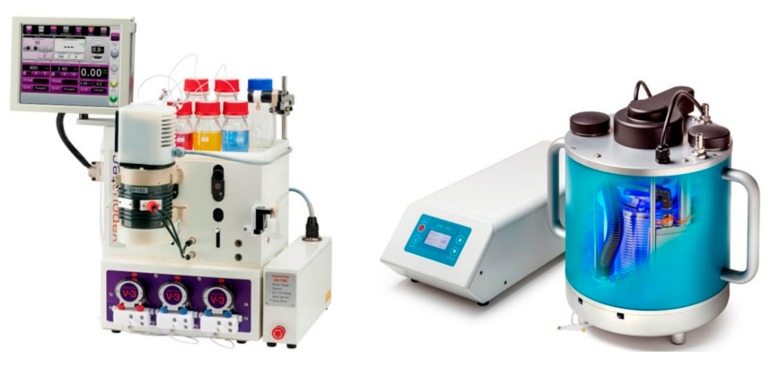
Images of the Vapourtec high-power LED reactor and Uniqsis’s PhotoSyn reactor.

In addition to the above photo-flow reactors that display innovative engineering to uniquely resolve limitations known from batch applications, a variety of photochemical reactors exist that are readily integrated with commercial flow modules. These are typically based on reactor coils made of various fluorinated polymers that are combined with suitable light sources ranging from LEDs to medium pressure Hg lamps. Examples of these systems include Vapourtec’s UV150 and high-power LED reactor (e.g., [32,33,34]) system and Uniqsis’s PhotoSyn (e.g., [35,36]), as depicted in Figure 6. Due to their modular nature, these systems are easy to use in everyday lab applications, and can be complemented with broad-band filters or dedicated photo-spectrometers. The popularity and effectiveness of these continuous flow photoreactors can be seen in many applications that have been reported over the years.

As can be seen from this compilation of continuous photo-reactors, numerous reactor types have been developed, and their successful application to different types of chemical reactions (i.e., cycloadditions, photooxygenations, etc.) demonstrates how limitations inherent to batch photochemistry can be overcome by appropriately engineered devices. All these systems have short pathlengths in common that result from thin films or small tubing diameters, and thus enable the effective and uniform irradiation of molecules. In addition, continuous flow processing ensures homogeneous irradiation profiles without over-irradiating substrates that might otherwise lead to undesired side reactions and accompanying discoloration of reaction products.

## 3. Greener and More Sustainable Approaches

Photochemistry is recognized as a valuable means for making new molecules, not only because it complements traditional thermal reactions, but also as it utilizes photons as cheap and readily available reagent equivalents. As such, photons may be viewed as traceless inputs, whose energy can be tuned based on their wavelength via Planck’s equation. Together with the notion that continuous flow chemistry may offer a more sustainable technology for chemical synthesis compared to traditional batch chemistry [37,38,39,40], this suggests that flow photochemistry is a green and almost ideal approach towards the synthesis of target molecules. In fact, photochemical transformations share this feature with electrochemical syntheses, as discussed in a recent comparative study [41]. While several articles have reported on the greener credentials of flow chemistry, and photochemistry potentially provides a cleaner means to making molecules, this section attempts to assess the remaining limitations of continuous flow photochemistry in this context.

One key to successfully performing photochemical reactions is the exploitation of appropriate light sources that emit photons of suitable wavelength to bring about a desired transformation. While traditional applications have been based on UV light, modern photochemical transformations such as those based on photoredox catalysis, commonly exploit LEDs. This change is desirable not only as it allows one to use lower energy light sources (≈10 W for LED versus ≈100 W for Hg lamp) that provide better selectivity, but also as considerably less heat is generated by LEDs. Consequently, using LEDs oftentimes avoids the need for high-power cooling systems, and concurrently minimizes the formation of thermal side-products. Arguably, the availability of LEDs delivering light of specific wavelengths contributes to more energy efficient chemical synthesis. In this vein, Ryu and co-workers reported in an early study on the energy efficiency of various light sources used in a typical [2 + 2]-cycloaddition reaction [42]. This comparative study evaluated a Hg lamp (300 W), a black light source (15 W) and an UV LED (1.7 W), and found that the LED not only gave the highest yield, but with a significantly higher energy efficiency.

**Figure 7:**
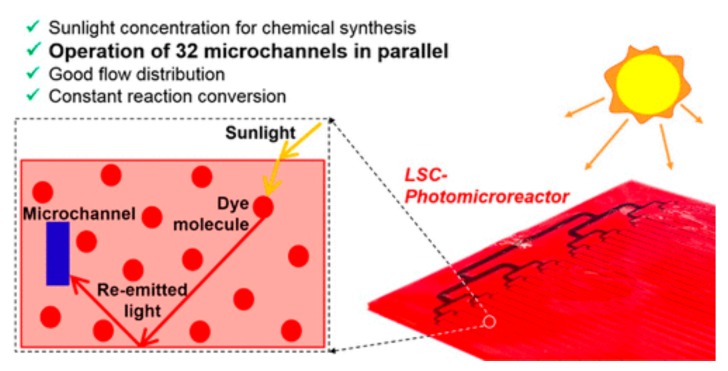
Exploiting solar light for scaled luminescent solar concentrators (LSC) applications (reproduced with permission from [48]).

Noël and co-workers [43,44] have recently described the exploitation of solar light in continuous flow settings. The use of solar light is highly attractive [45]—and in fact the first photochemical transformations were studied using sunlight [46,47] (many of which are associated with the pioneering works of Cannizzaro, Paterno and Ciamician); however, the heterogeneity of solar light (≈5% UV, ≈43% visible, ≈52% IR), together with inconsistent photon flux (seasons, cloud coverage, day/night pattern and geographical impact), make this approach challenging. In order to mitigate these challenges, luminescent solar concentrators (LSC, Figure 7) can be used to down-convert solar radiation to low-energy visible light that matches the absorption of specific photo-redox catalysts (e.g., methylene blue) which would then enable a given transformation. In such set-ups most of the incoming solar light is thus down-converted by fluorescent dyes embedded within the LSC, and transported within the device to microchannels where it is absorbed by a specific photocatalyst that triggers a chemical reaction. Although this technology is attractive, inevitable fluctuations in solar light input may require this approach to be coupled with conventional light sources to achieve a consistent photochemical process.

Besides the availability and use of suitable and energy-efficient light sources, a further challenge impacting the sustainability of photochemical reactions is the excessive use of solvents. An inherent problem in photochemistry is the requirement for high dilutions to favor productive interactions between photons and substrate (or catalyst) molecules. Therefore, most photochemical reactions are run at or below concentrations of 20 mM, which in turn results in the generation of copious amounts of solvent waste. It is therefore paramount to develop effective photochemical transformations based on non-hazardous solvents such as water, alcohols, acetone and ethyl acetate. Additionally, future continuous photoreactions should aim to incorporate solvent recycling systems whilst striving for processes that are tolerant to higher effective concentrations. Recent progress in this area has already highlighted the feasibility of performing multi-phasic photoreactions in slug-flow where suspensions of substrate and/or photocatalyst can be processed through microchannels. Micro-mixing can be achieved by Taylor flow in between immiscible phases (liquid/liquid, liquid/gas), which enhances the performance of such systems ([49]).

## 4. Synthesis of Bioactives: Drugs and Natural Products

As can be seen from the previous sections, several continuous photochemical approaches have been developed to overcome some of the intrinsic restrictions associated with light-driven reactions and subsequently applied to the synthesis of various compounds. Whilst this was in many cases directed towards establishing new or improved synthetic methods, this section will highlight some recent applications that have generated bioactive target compounds. This will showcase a selection of natural products, drugs and drug-like entities prepared in both academic and industrial laboratories.

**Figure 8:**
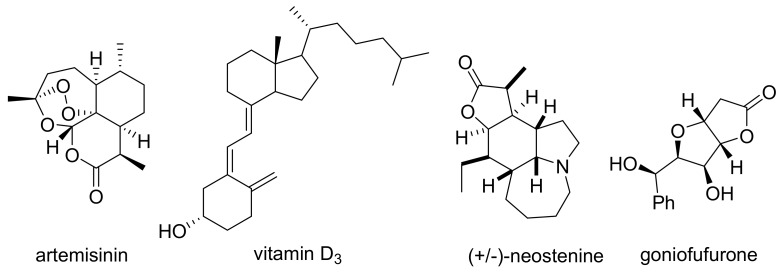
Structures of complex bioactive molecules featuring flow photochemistry key steps.

Early applications of continuous photochemical approaches oftentimes demonstrate the complexity of structures that can be realized in an effective manner. Several prominent examples, such as the generation of the anti-malaria drug artemisinin and its derivatives [50,51] and the preparation of vitamin D_3_ [52,53,54], outline the power of photochemistry when applied to the construction of unique targets. Further examples include the synthesis of neostenine [55] and goniofufurone [56], in which key bonds were realized photochemically, providing rapid access to advanced bioactive structures (Figure 8).

Other examples include the photochemical preparation of the anti-inflammatory drug ibuprofen by a photo-Favorskii rearrangement, which highlights an attractive and atom-economical continuous approach to this important target [57].

**Figure 9:**
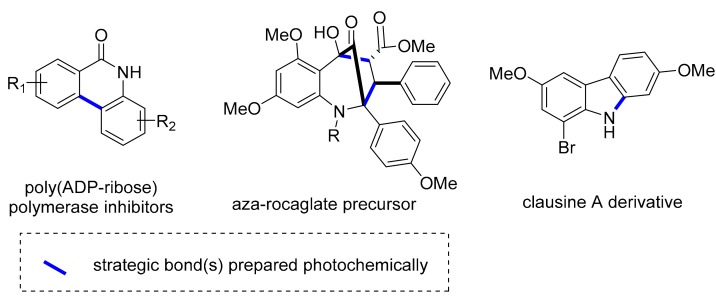
Structures of poly(ADP-ribose) polymerase inhibitors and natural product analogues prepared in flow.

More recent examples describe routes towards various poly(ADP-ribose) polymerase inhibitors aided by an intramolecular photocyclization reaction [58], unnatural aza-rocaglates via an excited state intramolecular proton transfer (ESIPT)-mediated (3 + 2) photocycloaddition [59] or the preparation of new derivatives of clausine A via an azide-mediated carbazole formation followed by an arylation reaction [60] (Figure 9).

**Figure 10:**
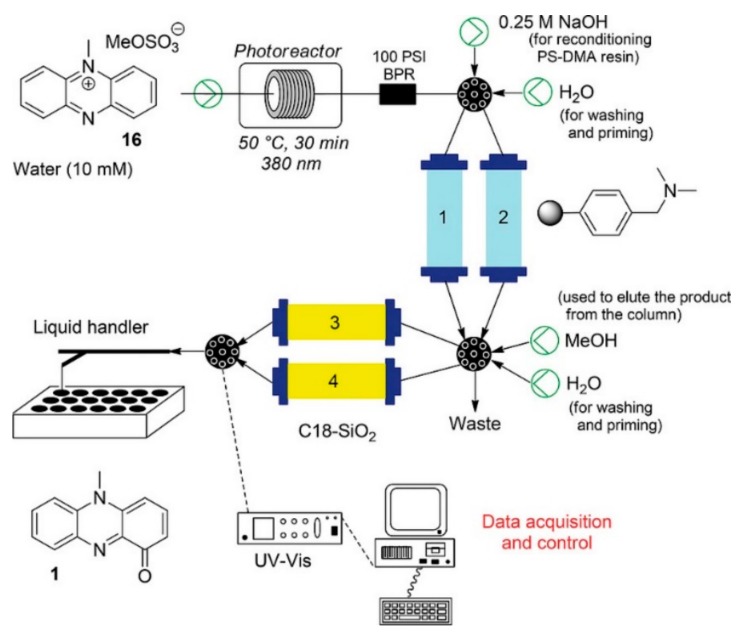
Continuous photosynthesis of pyocyanin (reproduced with permission from [61]).

Pyocyanin, a small naturally occurring virulence factor, was recently prepared in an effective and scalable multi-step approach featuring a photochemical oxygenation reaction (Figure 10). This process was, furthermore, coupled with immobilized reagents to facilitate the purification and isolation of the target product [61].

**Figure 11:**
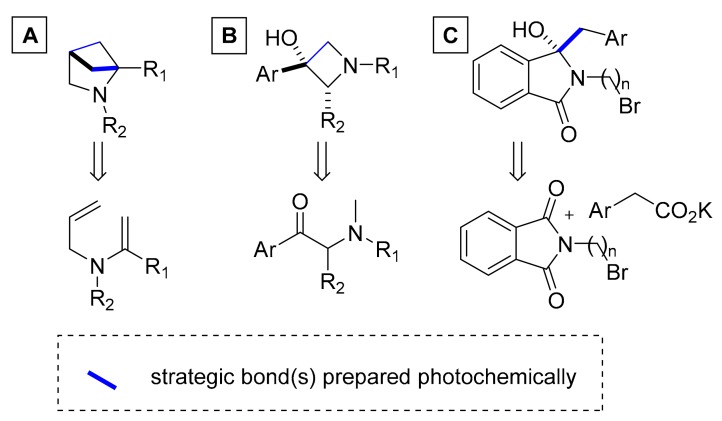
Structures of drug-like heterocyclic entities prepared photochemically and their precursors. (**A**) synthesis of 2,4-Methanopyrrolidines; (**B**) synthesis of 3-hydroxyazetidines; (**C**) photobenzylation of substituted phthalimides.

In addition to natural products and their analogs, several recent reports detail the use of continuous photochemical methods for the generation of drug-like heterocyclic scaffolds that hold interest in medicinal chemistry programs. 2,4-Methanopyrrolidines are an important heterocyclic class of compounds that display higher hydrophilicity than regular pyrrolidines. Several applications demonstrate that their continuous photochemical synthesis based on an intramolecular [2 + 2] cycloaddition (Figure 11A) can be scaled to kilogram quantities in order to facilitate further derivatization [31,62,63]. The synthesis of 3-hydroxyazetidines via a continuous Norrish–Young photocyclization reaction (Figure 11B) represents a further demonstration of effectively creating drug-like structures in a simple and atom-economical fashion, and a recent study reports on the versatility of this transformation [64]. A final example outlines the combination of photochemical with thermal processes in a reaction sequence rendering several isoindolin-1-one derivatives in a continuous manner, in which a photobenzylation of substituted phthalimides features as key step [65] (Figure 11C).

## 5. Remaining Challenges for Effectively Integrating Photoreactions within Multistep Sequences

The previous section outlined the breath of applications in which continuous photochemical transformations were effectively exploited to bring about the generation of bioactives in the form of drugs, natural products or their analogues. A variety of chemical transformations features in these syntheses ranging from cycloadditions and rearrangements to different radical-mediated sequences. In addition, photochemical flow approaches have been developed with great success for functionalization reactions, such as halogenations [66,67,68,69], trifluoromethylations [70,71,72], oxygenations [73,74,75] and C-H activations [76,77,78]. All these applications showcase how continuous set-ups help to overcome limitations commonly encountered with classical photochemistry, and it is apparent that a variety of light sources as well as reactor configurations are exploited to best serve the intended chemical route.

At this stage it appears that most of the highlighted studies were exploring the feasibility of photochemistry to achieve the improved flow synthesis of molecules of interest, which in many cases also addressed scalability to gram or even kilogram quantities based on tailored reactor designs. However, it is currently less common that such continuous photochemical syntheses are embedded within telescoped flow sequences in the same manner; this has been achieved for non-photochemical reactions [10]. Based on the advances presented in this review, it will take place; however, some bottlenecks will need to be overcome. As such, many photochemical reactions are still prone to generate multiple impurities, albeit in significantly lower amounts than in most batch applications. Currently, the effective separation of these impurities is typically achieved by off-line column chromatography rather than effective in-line purification tools. In addition, most cases reported in this review still rely on relatively high dilutions in order to realize effective photochemical transformations. While this will prevent formation of precipitates that can be detrimental to flow reactions, these high dilutions are unattractive, as they result in larger amounts of solvent waste. Furthermore, as the effective telescoping of additional downstream reactions requires matched concentrations of reactive species, various in-line analysis tools will be vital in achieving effective multi-step sequences [13,79]. Additional tools for unit operations that help with recycling solvents and concentrating streams in situ may be required both to improve the sustainability (E-factor and process mass index) and ensure the kinetics of bimolecular reactions are not hampered by exceedingly high dilutions.

Additional advances can be expected based on substituting high-power UV lamps with high-efficiency LEDs and utilizing solar light to bring about the photochemical synthesis of target molecules in a much more sustainable manner.

## 6. Conclusions and Outlook

Based on the applications highlighted in this short review, continuous photochemistry has indeed enjoyed a very successful period with numerous reports detailing its various benefits. From this, it is evident that the best solutions are obtained when innovative reactor designs are developed and applied to a given synthetic challenge, thereby exemplifying the synergy that can be gained from the union of synthetic chemistry and chemical engineering.

At this point it appears that most applications of continuous flow photochemistry target synthetic methodology based on photo-cycloadditions and photo-rearrangement reactions. However, several reports demonstrate the applicability of these methods to the generation of more complex structures, such as natural products and their analogs, which in many cases is complemented by more scalable processes to deliver gram or even kilogram quantities of material.

A further positive development in this field concerns the transition from non-selective UV lamps to more energy-efficient, high-power LEDs, and in some cases even solar light. This directly impacts the sustainability of the resulting processes and will be a key element in achieving greener chemical synthesis.

Whilst these developments were typically led by academic researchers, it is understood that all major pharmaceutical companies are developing their own continuous photochemical transformations. In addition, many publications outline collaborative efforts between academics and their industry-based colleagues, again highlighting the synergy of such joint projects. It can be anticipated that this development will render further applications of continuous light-driven reactions, enabling the synthesis of bioactive compounds, and as a consequence, will compensate for the relative scarcity of drug-based applications at this point through innovative and powerful applications in both medicinal chemistry settings and the scaled production of drugs.
